# Differential methods of localisation of fungal endophytes in the seagrasses

**DOI:** 10.1080/21501203.2016.1218966

**Published:** 2016-08-25

**Authors:** S. Raja, Pon. Subhashini, T. Thangaradjou

**Affiliations:** Centre of Advanced Study in Marine Biology, Faculty of Marine Science, Annamalai University, Chidambaram, India

**Keywords:** Fungal endophytes, localisation, sporulation, DGGE

## Abstract

Sections of three seagrass species (*Halophila ovalis, Cymodocea serrulata* and *Halodule pinifolia*) were assessed for endophytes based on differential staining using light and fluorescence microscopy method. Acridine orange and aniline blue detected endophytic fungi in 20% and 10% of the segments, respectively, whereas lactophenol cotton blue was more sensitive to detect the fungal hyphae in 70% of the segments. Hyphae were the principal fungal structures generally observed under the cuticle, within the epidermal cells, mesophyll (Parenchyma) cells and occasionally within the vascular tissue that varied in type, size and location within the leaf tissue. Present study also recorded the sporulation for the first time from the seagrass endophytes. Successfully amplified products of the ITS region of endophytic fungal DNA, directly from seagrass tissue and also from culture-dependent fungal DNA clearly depicted the presence of endophytic fungi in *H. ovalis* with two banding patterns (903 and 1381 bp) confirming the presence of two dominant fungal genera. The fingerprinting of endophytic fungal community within the seagrass tissue was assessed using denaturing gradient gel electrophoresis (DGGE) that derived with multiple bands that clarified the presence of more than one taxon within the seagrass tissue.

## 1. Introduction

The study of endophytes is generally regarded as a method-dependent process. To a greater extent, the fungal endophytic communities of plants are identified based on surface sterilisation technique, incubation conditions and sporulation, whereas the non-sporulating isolates (generally termed as sterile form) could only be identified by various molecular taxonomic tools (Guo et al. 2000) largely by culture-independent process (Guo et al. 2001). Due to the limitations in traditional sterilisation, isolation, culture and incubation techniques several endophytes are never isolated so far. As endophytes are internal and asymptomatic, their detection and identification are difficult, in general four sets of techniques such as (i) microscopy, (ii) isolation of pure cultures, (iii) biochemical and (iv) DNA-based methods (Bayman 2007) are used for their detection. All these methods are having their own advantages and disadvantages and the results are restricted to the choice of technique, and each may yield biased results in favour of certain taxa (Bayman 2007).

Surface sterilisation is insufficient evidence to ascribe an endophytic fungal location, and microscopy is considered as the only direct evidence (James 2000; Gyaneshwar et al. 2001) to ascribe the location. According to Kuldau and Yates (2000), light and electron microscopy can reveal the precise internal locations of endophytic fungi. Generally, assessments are made to determine whether the fungal infection is intercellular or intracellular and to establish the plant cellular anatomy. Staining techniques that aid in microscopic examination by differentiating hyphae from host tissues are fundamental to plant pathology, mycology and related disciplines (Hood and Shew 1996). Such staining techniques for examining the endophytic fungal assemblages in plant vary from simple single stain to complex multi-stain procedures. The purpose of this study was to develop an efficient method to detect the endophytic fungi in seagrass tissues with simple technical skill. Hence, three different stains acridine orange, aniline blue and lactophenol cotton blue were used in the study, so as to develop a simple and single staining method.

In parallel, DNA-based techniques have evolved which provide direct evidences for identification of dominant fungi within plant tissue and are not limited by cultivability or affected by contaminants (Duong et al. 2006). DNA-based methods allow researchers to explore the endophytic fungi, which are difficult to grow in culture media (Gotz et al. 2006) and also the non-sporulating sterile mycelia. PCR-based methods with specific primer pairs can provide an accurate, rapid and sensitive mean to detect and identify endophytic fungi within plant tissues (Guo et al. 2001). Hence, in the present study an attempt was made to identify and localise fungal endophytes in seagrass tissue using microscope, PCR-based and denaturing gradient gel electrophoresis (DGGE) methods.

## 2. Materials and methods

### 2.1. Localisation of endophytic fungi

Surface sterilised *Halophila ovalis, Cymodocea serrulata* and *Halodule pinifolia* samples were obtained from Palk Bay, India. Visual assessment of plant associated fungi was made by staining the seagrass tissues using a modified method of the Oses et al. (2008) and Alfaro et al. (2008). Randomly selected 90 (30 segments for each species) segments were stained using three different staining solutions: (i) acridine orange (0.05%), (ii) aniline blue (0.05%) and (iii) 1% HCl with lactophenol cotton blue (1:50, v/v) in order to identify the perfect stain for the detection of seagrass endophytic fungi. Surface sterilised leaf segments were transferred into 100 ml of double distilled water and incubated at 22°C in the light chamber for 48 hr. Autoclaved leaf segments were maintained as the control.

For staining with acridine orange and aniline blue, the seagrass tissue segments were fixed in a FAA fixative solution (10 ml of 40% formaldehyde, 5 ml of 99–100% glacial acetic acid, 50 ml of 99.9% ethanol and 35 ml of sterile distilled water). The leaf segments were stained using the above stain for 1 min. Acridine orange and aniline blue stained leaf segments were sectioned and examined under a fluorescence microscope (LEICA DM 2500) with green filter. For lactophenol cotton blue staining, inner epidermis of a leaf sheath was peeled off and placed on a glass slide and viewed the peeled side up. The samples were covered with cover glass and excess stain was then drawn off using tissue paper. Those slides stained using lactophenol cotton blue were observed using a light microscope (Magnus MLX-DX, Olympus Pvt. Ltd., India).

### 2.2. Culture-independent PCR-based method

#### 2.2.1. DNA isolation

Total fungal DNA was extracted from fresh surface sterilised tissue of the seagrasses by CTAB method of Gao et al. (2005) with slight modifications. Fungal DNA was extracted from cultured fungal strains by following the protocol of O’Donnell et al. (1997), by growing the seagrass endophytic fungal strains in 100 ml of potato dextrose broth at 28°C with constant shaking for three days. Genomic DNA of seagrass sample was extracted by the CTAB method described by Waycott et al. (2002).

#### 2.2.2. PCR amplification

The extracted genomic DNA served as a template for amplification of the ITS region of the nuclear DNA by using ITS1 (5′ TCCGTAGGTGAACCTGCGG 3′) and ITS 4 (5′ TCCTCCGCTTATTGATATGC 3′) primers. The PCR reaction was performed in thermal cycler (Techgene FTDENE 5D, England) with a red PCR master mix (Genei, Bangalore) by using the following programme, 3 min 95°C, 35 cycles of 40 s at 94°C, 50 s at 55°C, 60 s at 72°C, followed by 10 min of final extension at 72°C. The success of PCR reactions was checked by running 10 µl of PCR reaction on 1% agarose gel (40 mM Tris-acetate, 1 mM EDTA) with 1X TAE buffer and 0.1 µg of ethidium bromide per 1 ml of gel under 50 V applied current. DNA bands on the gel were visualised under UV light trans-illuminator. Images were made and stored in the Gel Doc system (Lark Gel Imaging system, India). The molecular weight determination was analysed by using the software of Lark Gel Imaging System.

#### 2.2.3. Denaturing gradient gel electrophoresis

DGGE test was conducted by following the method described by Tao et al. (2008) with minor modification. Electrophoresis was performed with 1 mm thick 8% polyacrylamide (acryl amide and bisacrylamide, 37.5:1) gel with vertical denaturing gradient of 40% urea and formamide for high denaturant with 15 µl TEMED. 10% APS was mixed for polymerisation and gel was run under 1X TAE buffer (40mM Tris, 40 mM acetic acid, 1 mM EDTA, pH 7.4). For DGGE, a total of 20 µl PCR product was mixed with the same volume of loading buffer dye (2% bromophenol blue, 50 mM tris buffer, 100% glycerol) and added to individual wells. Gels were run at a constant temperature of 58°C for 6–8 hr at 60 V and the temperature was controlled manually at 60°C by transferring warm buffer into the tank. Visualisation of the DNA bands was carried out by silver staining method.

## 3. Results

### 3.1. Localisation of endophytic fungi

The endophytic fungal development in seagrass tissues by the penetrating hyphae was observed using light and fluorescence microscopy. Ninety leaf segments of three seagrass species were assessed for fungal localisation based on differential staining (acridine orange, aniline blue and lactophenol cotton blue) and microscopic observation. The endophytes varied in type, size and location within leaf tissue. Hyphae were the principal fungal structures observed, generally found under the cuticle, within the epidermal cells, mesophyll (parenchyma) cells and occasionally within the vascular tissue. Particularly in lactophenol cotton blue staining method, hyphae were stained blue in colour and contrasted with the light green of the plant tissue.

**Figure 1.**
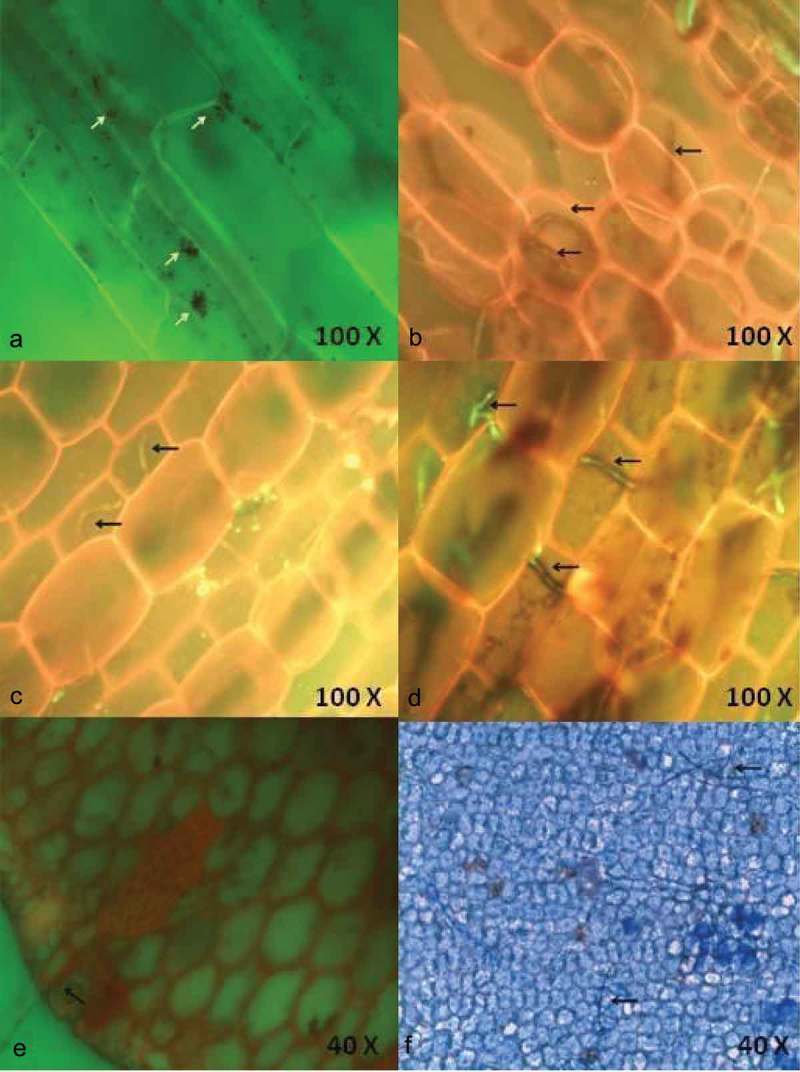
Localisation of endophytic fungi within seagrass tissue using 0.05% acridine orange (a-e) and Aniline blue (f) staining and the aero indicate the presence of fungal spores (a) and fungal hyphae in the cells.

**Figure 2.**
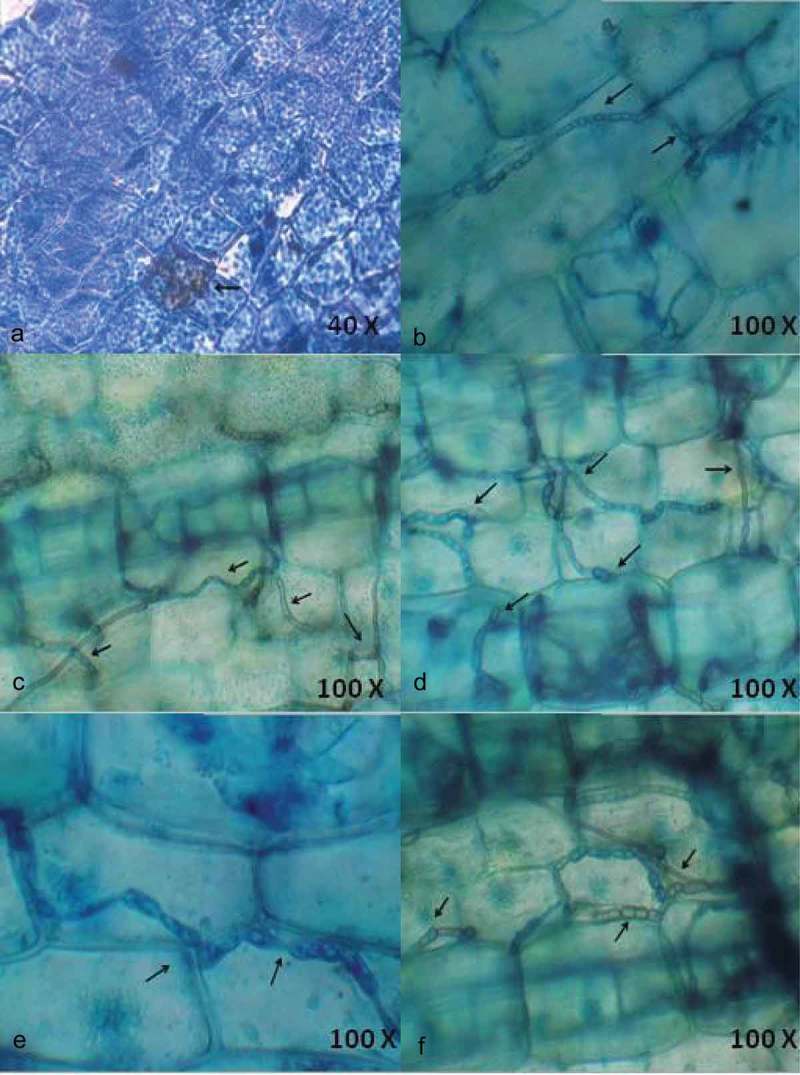
Localisation of endophytic fungi within seagrass tissue using Aniline blue (a) and lactophenol cotton blue (b-f) staining indicating the presence of septate fungal hyphae traversing the cells and forming network.

Among the three stains tested, acridine orange and aniline blue detected endophytic fungi in 20% and 10% of the segments, respectively, whereas lactophenol cotton blue was more efficient in detecting the fungal hyphae in 70% of the segments. The spores of the endophytic fungi were visualised clearly using acridine orange staining. Single hyphae and its growth were revealed in Figure 1(a-d), in which the apical elongation within parenchyma cells was visualised using 0.05% acridine orange. The hyphae penetrating outwardly from the fibrillar bundle of the rhizome was also visualised (Figure 1(e)). Aniline blue staining revealed the colonisation of fungal hyphae within the inner epidermal cells (Figure 1(f) and Figure 2(a). Among the three seagrasses visualized for endophytes, maximum fungal hyphae were observed within *C. serrulata* when compared to the other two species.

**Figure 3.**
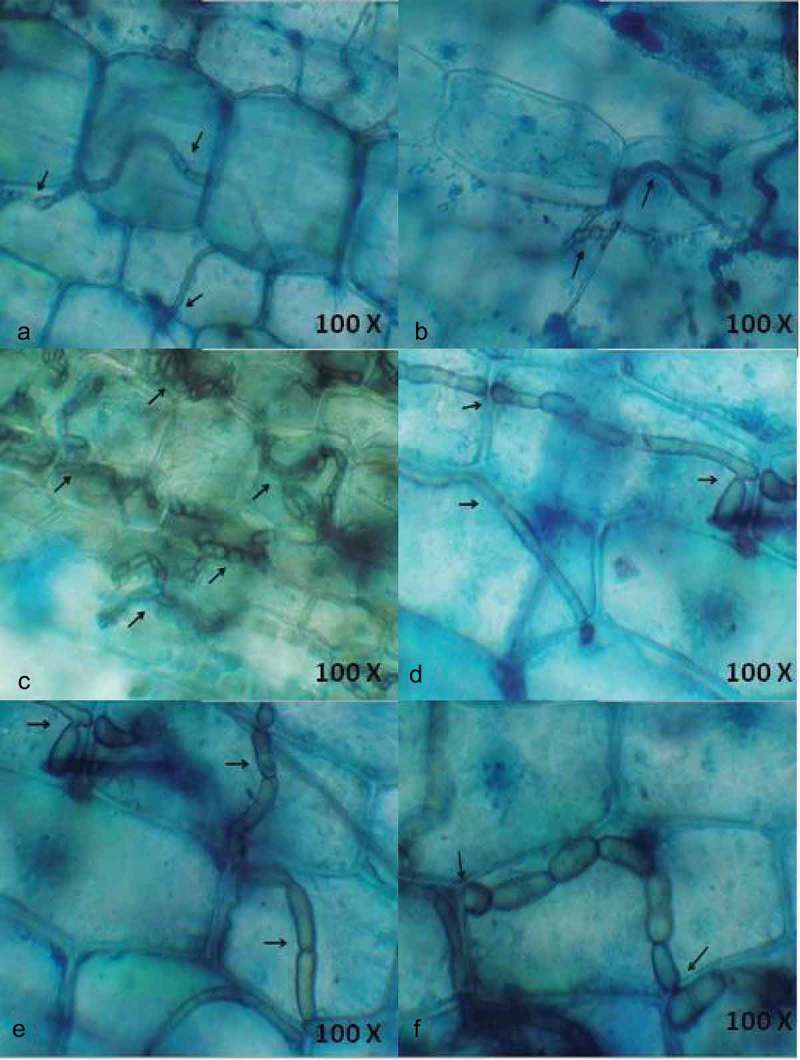
Localisation of endophytic fungi within seagrass tissue using lactophenol cotton blue (a-f) staining, closer view of septate fungal hyphae.

Interestingly, lactophenol cotton blue stain was more effective in staining the net-like hyphae (Figure 2(b-f)). The entry of hyphae from one epidermal cell to another and the clustering of hyphae within a parenchyma cell were also observed (Figure 2(b)). Figure 2(c-f) depicts the entry of hyaline hyphae into adjacent parenchyma cells. This denotes the ability of hyaline hyphae to absorb the blue staining. The extensive colonisation of dark septate endophytes (DSE) was observed in the parenchyma cells (Figure 3(a-f)). Large sclerotium-like structure was detected in parenchyma cells (Figure 3(d-f)) and small sclerotium-like structure was found in Figure 3(a-c) representing the different growth stages of DSE. DSE observed within the cells are capable of growing through the adjacent cells and extend outside the tissue. It was observed that the hyphae moved between the parenchyma cells. The result confirms the location of endophytic fungal colonisation in seagrass tissues.

### 3.2. Culture-independent PCR-based method

Three different DNA extraction methods were employed in this study for the isolation of fungal DNA directly from seagrass tissue, from cultured fungal strain and genomic DNA from seagrass. All the three methods resulted in good DNA quality that showed intact bands in agarose electrophoresis, and were chosen for PCR amplification. The PCR amplifications were successfully performed with the total DNA extracted from seagrass tissue and also from fungal strain.

**Figure 4.**
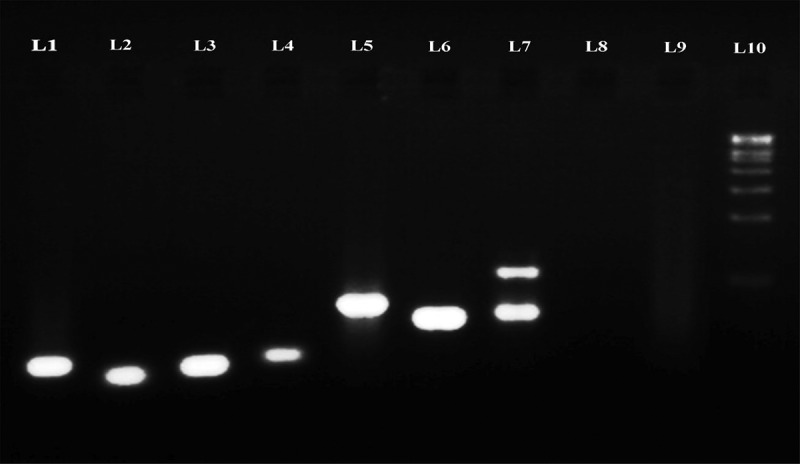
Agarose electrophoregram profile of ITS sequences of endophytic fungal DNA directly from seagrass and cultured fungal DNA amplified using ITS1–ITS4 primers.

**Figure 5.**
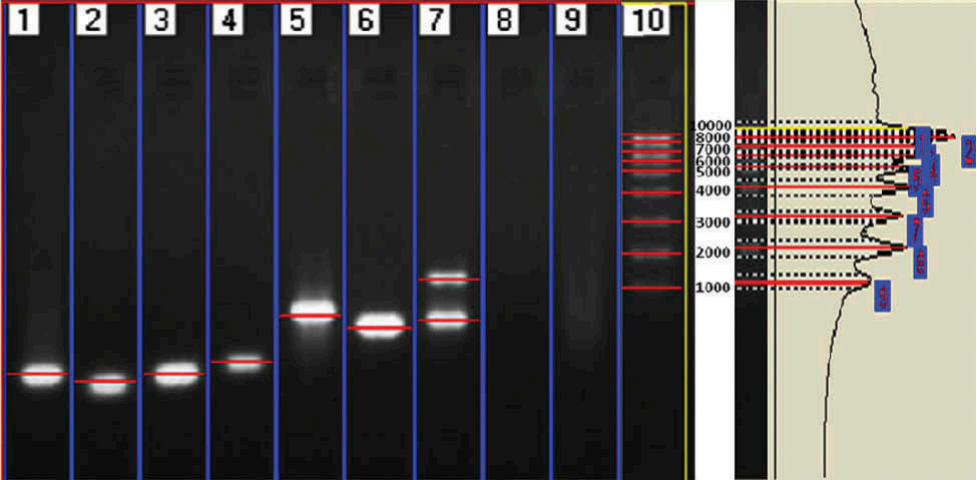
Electrophorogram showing the molecular weight pattern of the amplified PCR products.

The electrophorogram (Figure 4) of the amplification products clearly depict the presence of endophytic fungi in seagrass leaf tissue and culture-dependent fungal DNA. The number of base pairs of the amplification products was determined using Lark Gel imaging system and presented in Figure 5 and Table 1. The uncultured endophytic fungi from *H. ovalis* (Lane 7) showed two banding patterns (903 and 1381 bp), which confirmed the presence of two dominant fungal genera. However, *C. serrulata* (Lane 5) and *H. pinifolia* (Lane 6) showed a single band (855 and 852 bp, respectively). Similar to that, cultured endophytic fungal strains (Lane 1–4) revealed specific band which confirmed the amplification of ITS region, ranging from 519 to 608 bp for each strain, whereas the seagrass genomic DNA (Lane 8 and Lane 9) was not amplified using the fungal primer confirming the specificity of the primer for fungal DNA alone.

**Table 1.** Molecular banding patterns of the PCR amplified products in endophytes, un-cultured and seagrass samples.

Lane	Sample ID	DNA Source	Molecular weight
L1	SGE 26	Culture-dependent	563 bp
L2	SGE 27	Culture-dependent	519 bp
L3	SGE 24	Culture-dependent	563 bp
L4	SGE 21	Culture-dependent	608 bp
L5	SGE-UC1	Culture-independent	855 bp
L6	SGE-UC2	Culture-independent	852 bp
L7	SGE-UC3	Culture-independent	1381 and 903 bp
L8	SG1	Seagrass	Not amplified
L9	SG2	Seagrass	Not amplified
L10	Marker	–	1 kb

SGE: Seagrass Endophytes; UC: Un Culture; SG: Seagrass

### 3.3. Denaturing gradient gel electrophoresis (DGGE)

**Figure 6.**
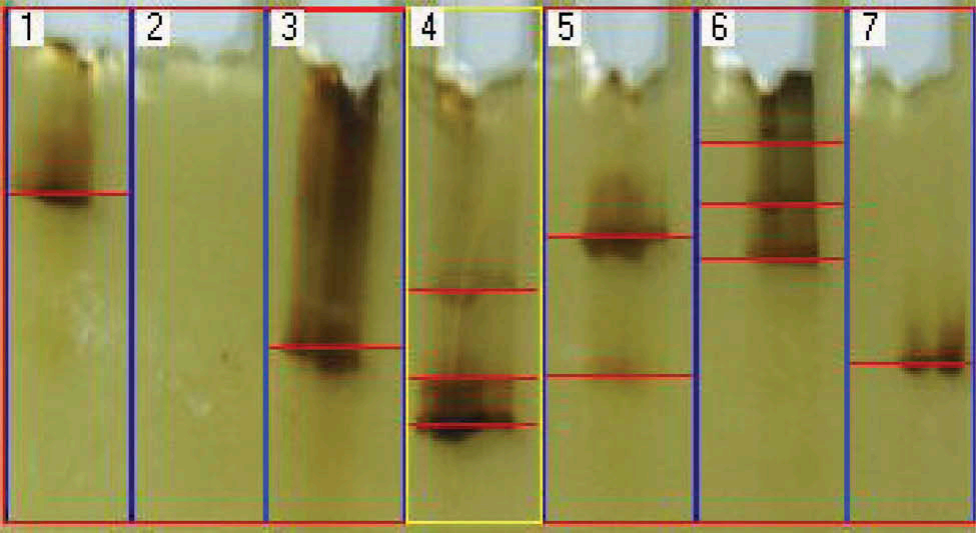
Amplified fungal DNA from culture-dependent endophytic fungi (Lane 1, 3 and 7), culture-independent endophytic fungi (Lane 4, 5 and 6) and seagrass (Lane 2) separated by DGGE.

DGGE patterns of seagrass endophytic community were composed of 1-3 bands. These numbers only include bands of ITS rRNA gene PCR products amplified from the seagrass tissue. The PCR product was visualized in 6 lanes out of 7 lanes loaded (Figure 6). Lane 1, 3 and 7 were cultured strains of SGE 21, SGE 24 and SGE 26, respectively, whereas Lane 4, 5 and 6 were SGE-UC1, SGE-UC2 and SGE-UC3. Lane 2 was loaded with the seagrass genomic DNA. Lane 1, 3 and 7 exhibited single band of the culture-dependent fungal DNA. At the same time, Lane 4, 5 and 6 loaded with culture-independent DNA showed more than two DNA band pattern. Lane 4 was loaded with the whole fungal DNA sample from *C. serrulata* (SGE-UC1), Lane 5 was loaded with that of *H. pinifolia* (SGE-UC2) and Lane 6 with *H. ovalis* (SGE-UC3). No band was found in Lane 2 as the ITS primer used in the present study was not capable of amplifying seagrass DNA. More than 2 banding patterns of DGGE in Lane 4, 5 and 6 expresses more than two fungal communities inhabiting the seagrass tissues. As the detection of fungal communities only considered in the present study, the standard markers were not used to analyse the range of DNA.

## 4. Discussion

The endophytic fungal community residing within the seagrass tissues was successfully investigated using microscopical and PCR-based methods. Light microscopy evidenced the presence of endophytic fungal hyphae within the cells of seagrass tissue and thus allowed to understand the colonisation pattern of endophytic fungi. Techniques of staining, light microscopy and scanning electron microscopy detect the presence of fungal structures in the hosts (Garcia et al. 2012) clearly. The present study founds evidence for the endophytic colonisation within seagrass tissue using three different staining methods through the visualisation of the hyphae. It is well known that the histological observations of hyphae provide additional evidences for the presence of endophytic fungi (Spurr and Welty 1975) and also indicate the exact location of colonisation. The growth of endophytic fungi within different cells of the tissue is evident from the net-like hyphae penetrating through the cell wall and extending from the fibrillar bundle and parenchyma cells. Microscopical methods can reveal the precise locations of endophytic fungi (Kuldau and Yates 2000), and the extent of colonisation (Muniz et al. 2011) as evident in the present study also. But the microscopical studies are not enough to go up to the identification and differentiation of various fungal forms inside the tissue for which we need to go for culture-dependent and -independent methods.

Different staining procedure for detecting the fungi from different plants are reported earlier (Petrini and Fischer (1990); Kowalski and Kehr (1992); Baum et al. (2003); Gomez-Vidal et al. (2006); Oses et al. (2008); Bernardi-Wenzel et al. (2010); Garcia et al. (2012)). However, there is a dearth of knowledge of microscopical studies of endophytic fungal assemblage within the tropical seagrass.

Acridine orange, a fluorescent stain has been used for staining nucleoproteins and tissue mucopolysaccharides. This stain has proved to stain endophytic fungi within plant tissue sections which gets fluoresced under blue–violet light. This stain has been used a lysosome marker in fungal spores (Wilson et al. 1978), and is one of the vital dye that has been used for a long time for a variety of cytological determinations. Aniline blue fluorescence has been widely used in plant histochemistry and also been applied in the study of plant fungal interactions (Stanghellini et al. 1993; Hood and Shew 1996). Aniline blue binds with various glucans and polysaccharides of plants and fungi. Lactophenol cotton blue stain with the higher concentration of phenol content deactivates the lytic cellular enzymes and thus the cells do not lyse. This acidic dye stains the chitin in the cell walls of fungi. Among the staining methods, acridine orange exhibited the fluorescent nature of the fungal hyphae, while aniline blue showed dark blue patches of the hyphae. Of all the three, lactophenol cotton blue derived the margins of hyphal structures very clearly in dark blue colour.

The present result portrays the efficiency of the lactophenol cotton blue stain in observing extensive and disseminated colonisation along all over the leaf, in the parenchyma and adaxial epidermis, indicating close interaction among the endophytes in multiple structural and trophic subniches in the host (Bernardi-Wenzel et al. 2010). A gradual increase of fungal colonisation and proliferation was clearly observed with lactophenol cotton blue staining. In the seagrass tissue, the fungus colonises and completely fills up single cells before it penetrates the adjacent cells. Whereas an unrestricted net-like intra and intercellular colonisation pattern was also observed. Interestingly, fungal spores were visualised within the seagrass cells for the first time. Abundance of fungal hyphae observed using the chitin staining was the same as that of using lactophenol cotton blue (Sathe and Raghukumar 1991), which symbolises that lactophenol cotton blue is the ideal stain to detect fungal endophytes within the seagrass tissue.

Microscopic observation of the stained seagrass leaves revealed various hyphal structures (Figure 1) that indicated the presence of different fungal communities. No such work on the localisation of endophytic fungi using microscopical methods has been reported earlier from seagrasses. Fine thread-like intracellular hyphae were observed in using all the three staining methods traversing the walls of the cells. This observation is found similar to the presence of extremely fine intracellular hyphae (0.5 µm) traversing the cell walls in the detritus of the seagrass *Thalassia hemprichii* (Sathe and Raghukumar 1991). In general, *C. serrulata* leaves have dense hyphae of endophytic assemblages rather than other seagrass species emphasising higher assemblage of endophytic fungi. This might be due to the broader leaf structure of *C. serrulata* when compared with *H. pinifolia* and *H. ovalis*, since more endophytic fungi are present in the longer and wider seagrass leaves (Wilson 1998; Sakayaroj et al. 2010).

In lactophenol cotton blue staining, extremely fine intracellular hyphae were observed occasionally and more often closely dark septate hyphae of different width were present within the leaves. Many of the hyphae were stained similar to the cell wall contour and were intercellular displaying distinct T-shaped branching. The hyphae growing within the cells showed neck-like structures near to the cell wall where the fungus traverses a cell wall. Occasionally, subepidermal hyphae penetrated the space between the cell and plasma membrane of cortical cells. The penetration of the fungal hyphae in the cell walls was clearly visible, especially the distribution of fungus between the neighbour cells evidencing the sites of penetration and invasion. This indicates that cell walls, complex carbohydrates are easily broken by its enzymatic apparatus (Muniz et al. 2011). It possibly means that the penetration of the fungus into the cells is related to the growth and expansion of the mycelium. The hyphae were frequently swollen at the site of branching forming sclerotium-like structures. A similar observation was made by Sathe and Raghukumar (1991) which proves that the fungal entities within the seagrass tissue exhibit more or less same characters.

Interestingly, DSE fungi were observed very clearly using the lactophenol stain. The melanised hyphae, typical for DSE fungi, are considered to be of importance for the host to survive stress conditions because cell wall melanin can trap and eliminate oxygen radicals generated during abiotic stress (Yuan et al. 2010). This gives a clear idea about the abiotic stress tolerance exhibited by the seagrass are accompanied by the role of endophytic fungi by trapping the free oxygen radicals. Light microscopy and trypan blue staining identified the presence of hyphae and mycelial mass in the cells, in intercellular spaces and crossing from one cell to another (Garcia et al. 2012) of *S. saponaria*. The data presented here also portrays the inter and intracellular hyphae of different diameters in the parenchyma cells, showing an extensive spread of hyphae both transversely and longitudinally. The importance of detecting inter or intracellular colonisation by fungal endophytes is to better understand endophyte/host interaction (Garcia et al. 2012).

The intracellular colonisation of leaves by fungal endophytes indicates a high interaction between them, positively selected in co-evolutionary event. The present work allowed us to visualise how and where fungal endophytes inhabit and spread through the cells of apparently healthy seagrasses. Fungi found in intercellular spaces caused no apparent damage to the cells by the hyphae. Microscopic analyses demonstrated a fungal colonisation pattern with the developmental stage of the seagrass tissue. The smaller sized cells exhibited hyaline hyphae whereas the matured cells exhibited wider hyphae. Mostly, the fungal colonisation takes place by intra and intercellular invasion of surrounding tissue and gradually increases with maturation of tissues. Colonisation initiates from chlamydospores which upon germination finally form a hyphal network inside the tissue. Hyphae enter the subepidermal layer through intercellular spaces where they branch and continue to grow (Deshmukh et al. 2006). Hyphal colonisation was predominantly observed within the intra and intercellular spaces of parenchyma cells. These results demonstrate the endophytic colonisation of seagrass leaves indicating a process of intimate interaction between the endophyte and the host plant. According to Kuldau and Bacon (2008), the intracellular spaces actually have many organic and inorganic nutrients, able to support the concentration of endophytic fungi.

The microscopical evidence was well supported by the molecular assessment made in the study. PCR-based technique confirmed the results obtained through microscopic examination for the presence and colonisation of endophytes. Culture-independent molecular techniques have been proved useful for evaluating endophytic communities in plants (Guo et al. 2001). Compared with the traditional culture techniques, the DNA-based methods may have advantage to identify microorganisms that are difficult to culture *in vitro* (Gao et al. 2005). However, the reliability and reproducibility of molecular techniques have to be ensured by the technical factors like efficiency of DNA extraction, PCR biases and selection of clones (Tabatabaei et al. 2009).

The three DNA extraction methods used for the isolation of DNA from different sources (culture-independent fungal endophyte, culture-dependent fungal endophyte and seagrass) resulted with good quality and quantity of DNA suitable for PCR amplification. Though the total DNA extracted from seagrass exhibited little shearing during initial extraction and the method was further modified as described here and resulted with good DNA by avoiding protein and other cellular contaminants. The PCR amplifications were successfully performed with the total DNA extracted from seagrass tissue, cultured fungal and seagrass isolated DNA. Since, fungal rDNA fragments were amplified from the total DNA sample with the method described in the present study, which resulted consistent and reliable in fungal DNA extraction from seagrass tissues.

The universal fungal primers used, ITS1 and ITS4 were found efficient in amplifying the culture-independent DNA (Lane 5, 6 and 7 in Figure 2) isolated from fresh seagrass tissues and the culture-dependent fungal strains (Lane 1, 2, 3 and 4 in Figure 2). On the other hand, the seagrass genomic DNA was not amplified (Lane 8 and 9 in Figure 2) proving that the primer pairs ITS1/ITS4 can efficiently be used for the detection of endophytic fungi in seagrasses and their specificity in amplifying fungal DNA. Similarly, Guo et al. (2001) too reported that the primers ITS1 and ITS4 were more specific to fungi than to the host plant. Contrary to this, Gao et al. (2005) have reported the limitations of primer pair ITS 1/ITS 4 in amplifying several fungi and stressed up the use of different primer pairs so as to ensure the identification of all endophytic fungal community in the tissues. Interestingly, in the present study, the endophytic fungal DNA were only amplified using the primer pairs ITS1/ITS4 and this may be due to the PCR programming which was suitable only for fungal DNA and not for the host.

The banding pattern of the total DNA amplified from *H. ovalis* was different from *C. serrulata* and *H. pinifolia*. This illustrates that the endophytic community within seagrass varies between species. The dominant fungal genera residing in the tissues getting amplified, which is confirmed from the electrophorogram (Figure 4). The molecular weight pattern of the culture depends on SGE 26, SGE 27, SGE 24 and SGE 21 was different from the culture-independent endophytes SGE-UC1, SGE-UC2 and SGE-UC3. The culture-independent isolates exhibited higher base pair composition when compared with the culture dependent which symbolises that the isolates belonged to different taxa. This result confirms that both culture-dependent and culture-independent isolation methods have to be employed so as to understand the actual endophytic fungal assemblages in a seagrass tissue. Results of endophytic communities obtained with traditional and molecular techniques by Guo et al. (2001) indicates the presence of some taxa using both techniques, whereas others are more amenable to either one of these two techniques. This emphasises the need of a combined method to study the endophytic population.

The fingerprinting of endophytic fungal community within the seagrass tissue was assessed using DGGE. Fingerprinting methods are designed to allow for the rapid comparison of samples, identifying any similarities or differences in composition (Valaskova and Baldrian 2009). DGGE of PCR amplified material, screens out fragments up to 800 bp in length that confirms the presence of endophytic fungi. The DGGE electrophorogram (Figure 5) derived with multiple bands in Lane 4, 5 and 6, those loaded with total DNA from seagrass tissue (culture-independent DNA), whereas the culture-dependent DNA samples derived single band in Lane 1, 3 and 7. This clarifies the presence of more than one taxa within the seagrass tissue. Though the results obtained revealed the localisation of fungal community within the seagrass, it was found low when compared with earlier reports (Gotz et al. 2006). The most important confirmation in the DGGE analysis is whether just the presence or absence of bands or their intensities should be used (Valaskova and Baldrian 2009). DNA fragments of different sequences may have similar mobilities in the polyacrylamide gel which means a single band may not necessarily represent a single species (Gelsomino et al. 1999) and one species may also give rise to multiple bands because of multiple genes with slightly different sequences with different base pairs. Despite these facts, molecular techniques circumvent the cultivation problem and have become a powerful tool for the identification of non-sporulating morphospecies (Wang et al. 2005). Moreover, this technique also helps in confirming the presence of endophytic fungal forms inside the tissue without any ambiguity, so this can be used as a confirmation tool and also for studying the localisation of endophytic fungi qualitatively.

Molecular techniques carried out in the present study appeared to be complementary, but the detection of the endophytic fungi, particularly those that cannot be incubated in artificial media can directly amplified from the seagrass tissues. But there are certain limitations that are to be considered during molecular approach. However, the recent improvements in DNA extraction, PCR and molecular techniques made this technically accurate, rapid and sensitive for identification of endophytic fungi directly from plant tissues. Though DGGE has been used to assess bacterial communities within seagrass sediments earlier, the data from the present investigation is a preliminary one on tropical seagrass which forms an evidence for the assemblage of endophytic fungal community within the seagrass. Though DGGE is also providing a cutting edge application to reamplify the dominant individual bands which can be later cloned and sequenced to reveal the identity of the most prominent members of the fungal community (Muyzer et al., 2004). However, in the present study, it was aimed to localise the presence of fungal endophytes by both microscopic and PCR-based methods and not attempted to go up to the identification of the dominant forms.

Bougoure and Cairney (2005) stated that culture-based methods and culture-independent DNA methods were similarly efficacious in their abilities to identify the most abundant members of fungal communities. But the present data along with those of Allen et al. (2003), indicate that this is not always true and both have potential bias (Bridge and Spooner 2001; Anderson and Cairney 2004). Hence, a combination of both approaches should probably be adopted in all investigations of this nature so as to ensure the actual endophytic fungi in seagrasses.

Microscopical methods are the only available method to confirm the exact location of the fungal hyphae within the plant tissue and lactophenol cotton blue is identified as the best stain for visualizing fungal endophytes. Siting of sporulation within the host cells was recorded for the first time from seagrasses. PCR-based method and DGGE, the alternate culture-independent methods also confirmed the presence of fungal endophytes in seagrass tissues. The culture-independent methods are recommended as the best tool to study the endophytic fungal diversity in a holistic manner by including the uncultivable forms also. But, it also felt that some of the cultural forms are not tracked in such culture-independent methods. Similarly, the conventional microscopic evidence is essential to confirm the location of the endophytes.
